# A comparative map viewer integrating genetic maps for *Brassica *and *Arabidopsis*

**DOI:** 10.1186/1471-2229-7-40

**Published:** 2007-07-24

**Authors:** Geraldine AC Lim, Erica G Jewell, Xi Li, Timothy A Erwin, Christopher Love, Jacqueline Batley, German Spangenberg, David Edwards

**Affiliations:** 1 Plant Biotechnology Centre, Primary Industries Research Victoria, Department of Primary Industries, Victorian AgriBiosciences Centre, 1 Park Drive, Bundoora, Victoria 3083, Australia; 2 Victorian Bioinformatics Consortium, Plant Biotechnology Centre, Primary Industries Research Victoria, Department of Primary Industries, Victorian AgriBiosciences Centre, 1 Park Drive, Bundoora, Victoria 3083, Australia; 3 Division of Biomathematics and Bioinformatics, Rothamsted Research AL5 2JQ Harpenden, UK; 4 Australian Centre for Plant Functional Genomics, Centre for Integrated Legume Research and School of Land, Crop and Food Sciences, University of Queensland, Brisbane, QLD 4072, Australia; 5 Australian Centre for Plant Functional Genomics, Institute for Molecular Biosciences and School of Land, Crop and Food Sciences, University of Queensland, Brisbane, QLD 4072, Australia

## Abstract

**Background:**

Molecular genetic maps provide a means to link heritable traits with underlying genome sequence variation. Several genetic maps have been constructed for *Brassica *species, yet to date, there has been no simple means to compare this information or to associate mapped traits with the genome sequence of the related model plant, *Arabidopsis*.

**Description:**

We have developed a comparative genetic map database for the viewing, comparison and analysis of *Brassica *and *Arabidopsis *genetic, physical and trait map information. This web-based tool allows users to view and compare genetic and physical maps, search for traits and markers, and compare genetic linkage groups within and between the amphidiploid and diploid *Brassica *genomes. The inclusion of *Arabidopsis *data enables comparison between *Brassica *maps that share no common markers. Analysis of conserved syntenic blocks between *Arabidopsis *and collated *Brassica *genetic maps validates the application of this system. This tool is freely available over the internet on .

**Conclusion:**

This database enables users to interrogate the relationship between *Brassica *genetic maps and the sequenced genome of *A. thaliana*, permitting the comparison of genetic linkage groups and mapped traits and the rapid identification of candidate genes.

## Background

*Brassica *species represent important crops providing a major source of cooking oil, vegetables and condiments across many countries [1,2]. The species relationship of cultivated *Brassicas *was described by the "triangle of U"[3] with the three amphidiploid *Brassica *species *B. juncea *(AABB, 2n = 36), *B. napus *(AACC, 2n = 38) and *B. carinata *(BBCC, 2n = 34) formed through interspecific hybridization between the diploid *Brassica *species, *B. rapa *(AA, 2n = 20), *B. nigra *(BB, 2n = 16) and *B. oleracea *(CC, 2n = 18) [4,3]. *Brassicas *are closely related to the model species, *Arabidopsis thaliana*, for which the genome sequence was determined in 2000 [5]. Molecular genetic mapping has been applied for several *Brassica *species with identification of genomic regions and molecular genetic markers associated with heritable traits.

Genetic mapping in *Brassicas *has been predominantly based on Restriction Fragment Length Polymorphism (RFLP) and Simple Sequence Repeat (SSR) molecular markers [2,6-8]. In total, more than 900 different publicly available *Brassica *and *Arabidopsis *molecular markers have been mapped onto at least 15 genetic maps, usually derived from wide crosses, from European and Canadian cultivars.

Comparative mapping based on the alignment of chromosomes using common molecular markers helps researchers translate information from one map to another. Synteny between the genomes of different plant species was first characterised in grass species by Bevan and Murphy [9]. More recently, detailed comparisons within the Brassicaceae has demonstrated the practical value of comparisons between the genome of *Arabidopsis *and cultivated *Brassica *species [10]. This comparison permits the co-location of related traits from different maps and across different related species.

Previous comparisons between *Brassica *and *Arabidopsis *have identified significant regions of synteny and duplication. Lukens *et al. *[6] identified 34 significant regions between the *Arabidopsis *genome and a genetic map of *B. oleracea*, representing over 28% of the *B. oleracea *genetic map length. Long collinear regions are shared between *B. oleracea *linkage group (LG) 1 (O1) and *Arabidopsis *chromosome 5 (At Ch5), O5 and At Ch1, O3 and At Ch5, as well as several smaller regions of predicted synteny. In a more recent study by Parkin *et al. *[11], syntenic blocks were identified covering almost 90% of the mapped length of the *B. napus *genome. Each conserved block contained on average 7.8 shared loci and had an average length of 14.8 cM in *B. napus *and 4.8 Mb in *Arabidopsis.*

CMap is one of the more powerful tools for viewing and comparing genetic maps and has been applied successfully for comparison of genetic maps within and between related grass species [12,13]. We have applied this tool for the comparison of genetic maps from different *Brassica *species. Furthermore, we have identified candidate loci within the sequenced genome of *Arabidopsis *corresponding to *Brassica *genetic markers enabling the linkage between mapped *Brassica *traits and candidate genes in *Arabidopsis*. The *Brassica *comparative mapping tool is publicly available and integrated with a custom marker and trait database as well as an EnsEMBL based *Arabidopsis *genome viewer [14].

## Construction and content

CMap (version 0.13) was downloaded from Generic Software Components for Model Organism Databases (GMOD) [15] and implemented on an IBM × 335 server (2 × 2.8 GHz Xeon processors, 2 × 146 GB SCSI RAID5 and 4 GB RAM). *Brassica *molecular marker data was collated from various public sources. Marker correspondence was determined through nomenclature and sequence identity using WU-BLAST [16] with the following parameters, hspsepSmax = 1000, topcomboN 8, wordmask seg. Marker correspondence was also kindly provided by Isobel Parkin, Agriculture Canada. Candidate syntenic blocks are identified based on the definition of Parkin *et al. *[11]. A syntenic block requires a minimum correspondence between 4 mapped loci within 20 cM in *Brassica *to 4 regions within 4 Mb in *Arabidopsis*.

## Utility and Discussion

Currently the *Brassica *comparative map database hosts maps for *B. napus *(8), *B. oleracea *(7), *B. juncea *(5) *B. rapa *(3) and *Arabidopsis *(6). Molecular marker information was processed within the BASC MarkerQTL database as described in Erwin *et al. *[14], and genetic positions integrated within the comparative map database. A total of 834, 3499, 1740 and 860 markers were identified for *B. oleracea*, *B. napus*, *B. juncea *and *B. rapa*, respectively. No genetic map information was available for the species, *B. nigra *or *B. carinata*.

**Figure 1:**
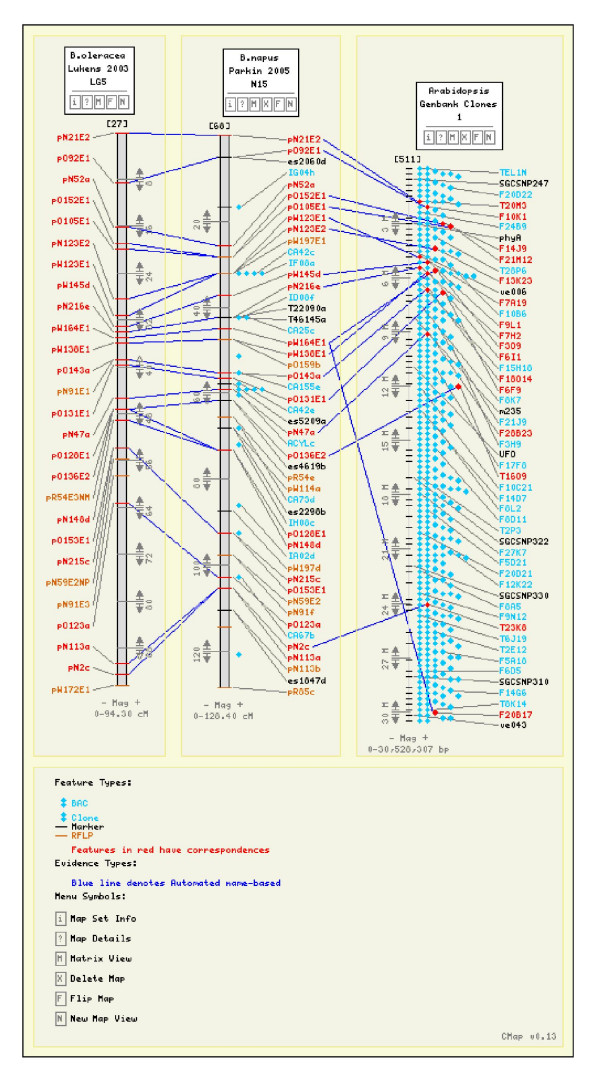
A comparison of *B. oleracea *Lukens *et al. *(2003) O5 map with *B. napus *Parkin *et al. *(2005) N15 and *Arabidopsis *Genbank Clones Chromosome 1.

Sequence information was available for 213 mapped SSR and 230 RFLP markers, enabling the prediction of corresponding loci on the *Arabidopsis *genome based on sequence identity. Candidate *Arabidopsis *positions for an additional 200 markers were generously provided by Isobel Parkin, Agriculture Canada. Correspondences between mapped markers and sequenced *Arabidopsis *BACs were collated from Lukens *et al., *[6]; Mayerhofer *et al., *[10]; and Suwabe *et al., *[17]. A total of 1116 markers correspond with 987 BACs, representing 238 markers *in B. oleracea *(28.5%), 777 markers in *B. napus *(22.2%), 44 markers in *B. juncea *(2.5%), and 281 markers in *B. rapa *(32.7%). In total, correspondence with *Arabidopsis *was determined for 16.1% of mapped *Brassica *markers. Corresponding markers between the different *Brassica *genomes and with *Arabidopsis *may be readily identified and viewed (Figure 1).

### Correspondence between *B. oleracea *markers and *Arabidopsis *BACs

A total of 1596 *Arabidopsis *BACs, represented *Arabidopsis *chromosomes 1–5. Of these BACs, 141 had corresponding *B. oleracea *markers. For At Ch1, 40 BACs were found to correspond to 75 mapped *B. oleracea *markers, of these 40 BACs, 15 (37.5%) of the BACs correspond to a single *Brassica *locus. Nineteen (47.5%) of the BACs correspond to two *Brassica *markers, while 6 BACs (15%) correspond to more than two *Brassica *markers. Of the 27 BACs from At Ch2 shown to correspond to 55 mapped *B. oleracea *markers, 13 (48%) of the BACs correspond to a single *B. oleracea *locus, 6 (22%) correspond to 2 *B. oleracea *markers, while 8 BACs (30%) correspond to more than 2 *Brassica *markers. Of the 26 BACs from At Ch3 shown to correspond to 47 mapped *B. oleracea *markers, 15 (34.6%) of the BACs correspond to a single *B. oleracea *locus, 19 (47.5%) correspond to 2 *B. oleracea *markers, while 6 BACs (15%) correspond to more than 2 *Brassica *markers. Twenty BACs from At Ch4 were found to correspond to 50 mapped *B. oleracea *markers, of these 7 (35%) correspond to a single *B. oleracea *locus, 4 (20%) correspond to 2 *B. oleracea *markers, while 9 BACs (45%) correspond to more than 2 *Brassica *markers. Of the 28 BACs from At Ch5 shown to correspond to 95 mapped *B. oleracea *markers, 12 (42.7%) of the BACs correspond to a single *B. oleracea *locus, 10 (35.7%) correspond to 2 *B. oleracea *markers, while 6 BACs (21.4%) correspond to more than 2 *Brassica *markers. The distribution of the BAC corresponding markers on each linkage group is shown in Table 1.

**Table 1:** Summary of correspondence between *B. oleracea *markers and *Arabidopsis *BACs, and the number and proportion of markers corresponding to each *B. oleracea *linkage group (O1–O9).

	At Ch1		At Ch1		At Ch3		At Ch4		At Ch5		Total
	No.	%	No.	%	No.	%	No.	%	No.	%	No.

O1	3	4	5	9	4	4	9	18	7	7.4	28
O2	3	4	7	12.7	5	4	2	4	19	20	36
O3	8	10.7	12	21.8	12	10.7	9	18	16	16.9	57
O4	9	12	13	23.6	5	12	7	14	9	9.5	43
O5	16	21.3	2	3.7	8	21.3	3	6	2	2.1	31
O6	8	10.7	4	7.3	2	10.7	2	4	3	3.1	19
O7	7	9.3	7	12.7	4	9.3	8	16	13	13.7	39
O8	12	16	2	3.7	4	16	4	8	3	3.1	25
O9	9	12	3	5.5	3	12	6	12	23	24.2	44

Total markers	75	100	55	100	47	100	50	100	95	100	322

### Comparison of *B. oleracea *with *Arabidopsis*

#### Arabidopsis Chromosome 1

**Figure 2:**
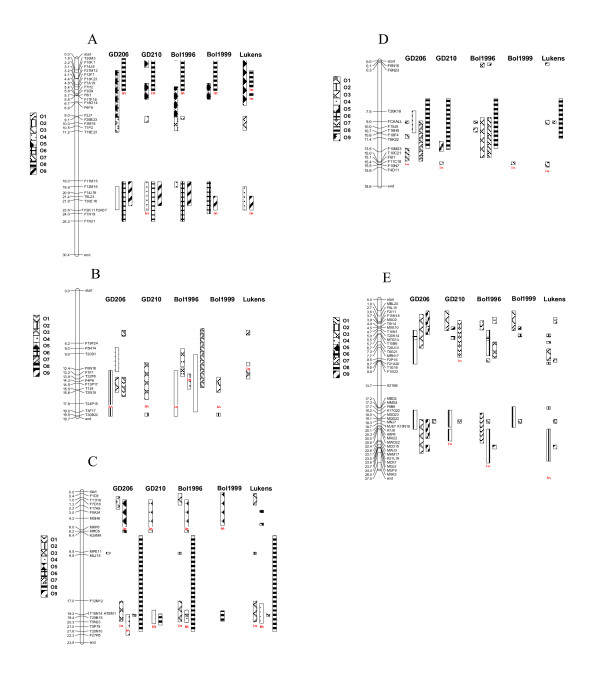
**A-E. **Comparison of the *B. oleracea *maps GD206 (A12XGD-206), GD210 (A12XGD-210), Bol1996 (BolAG_1996_A), Bol1999 (BolAG_1999_A) to *Arabidopsis *chromosomes 1–5.

The conservation between the different *B. oleracea *maps and At Ch1 was similar in most cases. No conserved blocks were identified between At Ch1 and linkage groups O1 and O2 (Figure 2a). A 2 Mb region was predicted to be syntenic to O3 in the A12XGD-206, BolAG_1999_A and Lukens *et al. *[6] maps, only 1 Mb of this region was syntenic with the A12XGD-210 O3, while there was no synteny predicted between At Ch1 and BolAG_1999_A O3. A 4 Mb conserved block, from the lower half of At Ch1, was predicted to be syntenic to O4 from A12XGD-206, A12XGD-210 and Lukens *et al. *[6], only 1 Mb of this region was syntenic with BolAG_1996_A O4, and the BolAG_1999_A O4 did not show any synteny. The 6 Mb conserved block predicted to be syntenic to O5 in all 5 maps was spilt in 2 in. A12XGD-210 and BolAG_1996_A and only 1.5 Mb was syntenic in BolAG_1999_A. A 5 Mb region in the lower half of At Ch1 was predicted to be syntenic to O6 of all but the Lukens *et al*. [6] map. Only a small conserved region is predicted to be syntenic to BolAG_1996_A O7. The 5 Mb conserved block predicted to be syntenic to O8 in all 5 maps, was only 3 Mb in Lukens *et al. *[6] map with an additional 0.5 Mb block. The 3 Mb conserved block predicted to be syntenic to A12XGD-206, A12XGD-210 and BolAG_1996_A O9 contained inversed markers in the middle of the BolAG_1999_A O9 syntenic block. Only 2 Mb of this region was syntenic in Lukens *et al. *[6] and BolAG_1999_A O9.

#### Arabidopsis Chromosome 2

No conserved blocks were identified between At Ch2 and linkage groups O1, O5, O6, O8 and O9 (Figure 2b). Similar sized conserved blocks were predicted to be syntenic to A12XGD-206 and BolAG_1996_A O2, however no synteny was detected in the other maps. The conserved blocks predicted to be syntenic to the A12XGD-206 map and BolAG_1999_A O3 were lengthened into 2 blocks in BolAG_1996_A O3. The syntenic region between At Ch2 and Lukens *et al. *[6] O3 was a smaller inverted conserved block in the same area. The 4 Mb block predicted to be syntenic to A12XGD-206 O4, spanned 10 Mb in BolAG_1996_A O4 with a further 3 Mb inverted block. The syntenic region in Lukens *et al. *[6] O4 was a 1 Mb inverted block. A 9 Mb conserved block in the middle of At Ch2 was predicted to be syntenic to BolAG_1996_A O7, a smaller region of A12XGD-206 O7 was also syntenic with two smaller blocks. No conserved blocks were identified in O7 from the other maps.

#### Arabidopsis Chromosome 3

The conservation between the different *B. oleracea *maps and At Ch3 was similar in most cases. No conserved blocks were identified between At Ch3 and linkage groups O1, O2, O6 and O9 (Figure 2c). The 2 Mb conserved block at the top of At Ch3, and 3 Mb inverted blocks at lower half of the chromosome were predicted to be syntenic to A12XGD-206, BolAG_1996_A and Lukens *et al. *[6] O3, with no synteny detected in the other maps. The 4 Mb inverted block predicted to be syntenic to O4 was similar in all maps, however not present in BolAG_1999_A. A 5 Mb conserved inverted block at the top of At Ch3 was predicted to be syntenic to O5 in all the maps except Lukens *et al. *[6] map, where only two 0.5 Mb blocks were detected. The 0.5 Mb conserved block predicted to be syntenic to A12XGD-206, BolAG_1996_A and Lukens *et al. *[6] O7 was not present in the other maps. Only 1.5 Mb of the 15 Mb conserved block, predicted to be syntenic to A12XGD-206, BolAG_1996_A and Lukens *et al*. [6] O8, was conserved in A12XGD-210 and BolAG_1999_A O8.

#### Arabidopsis Chromosome 4

No conserved blocks were identified between At Ch4 and linkage groups O2, O5 and O6 (Figure 2d). The only predicted synteny between At Ch4 and BolAG_1999_A was an inverted conserved block on O1, this was also present in A12XGD-210 O1. Three conserved blocks were predicted to be syntenic to A12XGD-206 and Lukens *et al. *[6] O1, with two of these blocks predicted in BolAG_1996_A. A 16 Mb conserved block at the lower half of At Ch4 was predicted to be syntenic to BolAG_1996_A O3, no blocks were detected in the other maps. Only O4 from A12XGD-206 was predicted to be syntenic with At Ch4. A 6 Mb conserved block in the middle of At Ch4 was predicted to be syntenic to A12XGD-206 and BolAG_1999_A O7, with a 0.5 Mb syntenic block detected in Lukens *et al. *[6] O7. The 6 Mb conserved block in the middle of At Ch4 was predicted to be syntenic to O8 in all the populations except BolAG_1999_A. A 2 Mb conserved block in the middle of At Ch4 was predicted to be syntenic to A12XGD-210 O9. There was also a small conserved block with between BolAG_1996_A and Lukens *et al. *[6] O9.

#### Arabidopsis Chromosome 5

No conserved blocks were identified between At Ch5 and linkage groups O4, O5 and O8 (Figure 2e). Only BolAG_1996_A O1 showed conservation with At Ch5. Two conserved block at the top and bottom half of A12XGD-206 map O2 were predicted to be syntenic to At Ch5. Two syntenic blocks were also found on the lower half of A12XGD-210 O2. Two blocks 3 Mb and 4 Mb were found at bottom half of BolAG_1996_A map. Two blocks were found on Lukens *et al. *[6] O2, a 2 Mb block was found in the lower middle of BolAG_1999_A O2 and two conserved blocks at the top and bottom half of A12XGD-206 O3 were also predicted to be syntenic to At Ch5. Two conserved blocks between At Ch5 and A12XGD-206 O3 were identified, a contracted version of one of these blocks was also syntenic in A12XGD-210 and BolAG_1999_A O3. One 2 Mb block was found in the lower top of BolAG_1996_A O3 and two blocks were found at the lower top of Lukens *et al. *[6] O3. A 0.5 Mb conserved block in O6 from all the maps was predicted to have a syntenic region At Ch5. A small conserved block in O7 in all the maps was predicted to have a syntenic region to At Ch5, with an additional 0.5 Mb block found in BolAG_1996_A and Lukens *et al. *[6] O5. Four conserved blocks between At Ch5 and A12XGD-206 O9 were identified. Three smaller blocks were conserved between At Ch5 and Lukens *et al. *[6] and BolAG_1996_A O9. Only a small conserved block in A12XGD-210 and BolAG_1999_A map O9 was identified.

### Comparison of *B. napus *with *Arabidopsis*

#### Arabidopsis Chromosome 1

**Figure 3:**
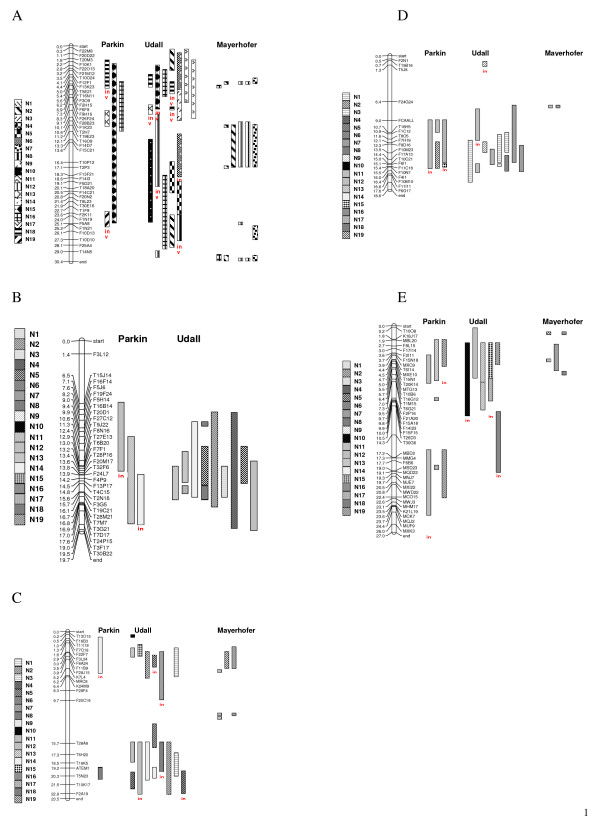
**A-E. **Comparison of the *B. napus *maps from Parkin et al. [11], Udall et al. [18] and Meyerhofer et al. [10] to *Arabidopsis *chromosomes 1–5.

No conserved blocks were identified between At Ch1 and linkage groups N1, N4, N11, N14 and N17. Using the mapping information from Parkin *et al. *[11], five conserved blocks were identified between At Ch1 and linkage groups N18, N13, N19, N15 and N16 (Figure 3a). The largest of these (N15) spanned nearly the whole of At Ch1, furthermore, the N18 region is inverted. The map described by Udall *et al. *[18] identified syntenic regions between At Ch1 and most linkage groups, with some inversions. The largest conserved regions matched N10 N16 and N12 (inverted), spanning ~10 Mb. The Mayerhofer *et al. *[10] map detected a 6 Mb block for N19, two for N12 and one for N7. Small blocks were found at the top and the bottom of the chromosome for N19, N12 and N7.

#### Arabidopsis Chromosome 2

The map from the Mayerhofer *et al. *[10] study did not identify any blocks to At Ch2. Using the mapping information from Parkin *et al. *[11] two inverted blocks for N12 and N14 were identified, along with a 6 Mb block for N13 (Figure 3b). The Udall *et al. *[18] genetic map had conserved blocks for N3, N5, N13, N14, and N18 with blocks larger than 3 Mb detected in N19, N4 and N12.

#### Arabidopsis Chromosome 3

No conserved blocks were identified between At Ch3 and linkage groups N1, N6, N8, N9, N11, N16 and N17. Using the mapping information from Parkin *et al. *[11] one 4 Mb inverted block from N14 at the top of At Ch3 and a 1 Mb block from N18 were identified (Figure 3c). Utilizing the information from the Udall *et al. *[18] map, conserved blocks in N2, N4, N5, N10, N12, N13, N14, N15, N18, and N19 were detected, with blocks larger than 4 Mb were found in N2, N3, N12 and N14. Inverted blocks were found in N4, N5, N7, N12, and N18. From the Mayerhofer *et al. *[10] map, three 0.5 Mb conserved blocks were found for N12 and 2–3 Mb blocks were found for N2 and N7 at the top of the chromosome.

#### Arabidopsis Chromosome 4

No conserved blocks were identified between At Ch4 and linkage groups N2, N4, N5, N8, N9, N10, N14, N16 and N18 (Figure 3d). Only two small conserved blocks were identified from the Mayerhofer *et al. *[10] map, on N7 and N12. Using information from the Parkin *et al. *[11] map, inverted blocks in N11 and N15 were identified. Further conserved blocks for N11, N17 and N19 were found in the middle of At Ch5. Utilising information from the Udall *et al. *[18] map showed inverted blocks for N11 and N13, spanning 3 Mb and 1 Mb respectively. Large blocks were found for N1, N3 and N7 in the lower half of the chromosome. Smaller blocks were also found for N11, N13, N3 and N6

#### Arabidopsis Chromosome 5

No conserved blocks were identified between At Ch5 and linkage groups N1, N3, N4, N5, N8, N9, N11, N14 and N18 (Figure 3e). Using the mapping information from Parkin *et al. *[11] inverted blocks were found for N10 and N17, spanning 6 Mb and 3 Mb at the top of At Ch5. Three conserved blocks larger than 3 Mb were found for N12, N13 and N19. A further 2 small segments for N17 were found in middle of the chromosome. Using the mapping information from Udall *et al. *[18] inverted blocks were found for N12 and N19, spanning 6 Mb and 3 Mb respectively, at the end of the chromosome. A 6 Mb inverted block was found for N6 in the middle of At Ch5 and. blocks larger than 4 Mb were found for N12, N13, N15, with a smaller block for N19 also at the top of the chromosome. Using the mapping information from Mayerhofer *et al. *[10] small blocks were found for *B *N2, N6, N12, and N16 at the top of At Ch5. A further large block was found for N7 at the top of the chromosome.

## Conclusion

We have collated available public *Brassica *genetic maps within a comparative mapping database and integrated this genetic and genomic information with the *Brassica *BASC MarkerQTL database and *Arabidopsis *EnsEMBL genome browser [14]. Known correspondences between *Brassica *markers and the genome of *Arabidopsis *were included to assist in comparative analysis between these species. Where possible, additional correspondences between *Brassica *loci and the *Arabidopsis *genome were identified using sequence identity. Due to genome duplication within *Arabidopsis*, *Brassica *markers frequently identified multiple candidate corresponding genome locations. To avoid losing potentially valuable correspondence data, up to the top three best matches (E < 0.00001) were included.

Blocks of genome conservation have previously been identified between the genomes of *Brassica *and *Arabidopsis *[6,11]. In this study we have compiled correspondences between *Brassica *and *Arabidopsis *from several different studies [10,11,15] and can thus compare how each of these maps correspond to the sequenced *Arabidopsis *genome.

Lukens *et al. *[6] identified 34 blocks of conservation between *Brassica *an *Arabidopsis*. The collinear regions identified in our study generally support this result. All 34 collinear regions were identified between the BolAG_1996_A map and the *Arabidopsis *genome, while the A12XGD-206, A12XGD-210 and BolAG_1999_A maps identified 28, 25 and 24 of the 34 regions respectively. None of these conserved collinear regions were identified between the NxG-97 map and *Arabidopsis *due to a lack of corresponding marker information. The difference between our results and the study of Lukens *et al. *[6] may be attributed to the method for conserved block identification. Lukens *et al. *[6] applied a statistical collinearity method and identified some small candidate conserved blocks (<1 Mb and 2.6 Mb). The method of Parkin *et al. *[11] applied in this study.

Parkin *et al. *[11] identified 21 conserved segments within the *Arabidopsis *genome which have been duplicated and rearranged to form the skeleton of the *B. napus *genome. However, not all of the marker BAC correspondences described by Parkin *et al. *[11] are publicly available, so we were unable to reproduce these results. In particular, many of the correspondences described between N1–N10 and *Arabidopsis *were not identified in our study. In addition, a lack of correspondence between *Arabidopsis *and the ends of *Brassica *linkage groups may be attributed to a low marker density in these areas.

Parkin *et al. *[11] suggests that the number of *Brassica *loci corresponding to each *Arabidopsis *chromosome was not evenly distributed. Fewer *Brassica *correspondences were identified to *Arabidopsis *chromosomes 2 and 3, with a greater number of correspondences identified to *Arabidopsis *chromosome 5 than expected. Our results from the analysis of collated *Brassica *data are in agreement with this finding. The results from our study of collated markers support this result. Previous studies have demonstrated that the *Brassica *genome is highly duplicated with suggestions of triplication compared to the genome of *Arabidopsis *[19]. Our analysis indicates abundant chromosome inversions, deletions and duplications resulted in a mosaic *Brassica *genome and supports the proposed hexaploid ancestor for the diploid *Brassica *progenitor [20]. A recent model for comparative analysis between the Brassicaceae suggest an ancient 8 chromosome karyotype [21]. As additional comparative Brassicaceae data becomes available, this model may be tested using these comparative genetic mapping tools.

This study has applied published and publicly available *Brassica *molecular marker and mapping information and collated this within a public *Brassica *comparative map database system, enabling the rapid retrieval, comparison and analysis of this information. There remain a significant number of studies for which sufficient data is not publicly available for inclusion in this system. The future inclusion of this data would further assist in the public understanding of *Brassica *genomes.

The linkage of *Brassica *genetic maps to the physical map of *Arabidopsis*, provides a resource where users may browse and search between the genome of *Brassica *or *Arabidopsis *and apply the knowledge gained from the study of this model plant for improvement in *Brassica *crop species. The genetic locations of traits identified within different maps and even different species may be compared. Candidate genes underlying traits may be identified through the linkage between genetic maps and the *Arabidopsis *EnsEMBL viewer. The completion of the genome sequence for *B. rapa *produced by the Multinational *Brassica *Genome Sequencing Project, along with the genome sequences for other related Brassicaceae will greatly assist in the characterisation of genome evolution in these species. The integration of this genome sequence information within the BASC *Brassica *database system [14] will provide the ability to link directly from *Brassica *genetic maps to the underlying candidate *Brassica *genes.

## Authors' contributions

GL collated the map data, performed the analysis of the data, created the figures and helped to draft the manuscript. EJ contributed to the collation of the map data and installation of the software. XL, TE and CL contributed to the installation of the software and critically reviewed the manuscript. JB contributed to the collation of the map data and critically reviewed the manuscript. GS critically reviewed the manuscript. DE conceived of the study and drafted the manuscript. All authors read and approved the final manuscript.

## Availability and Requirements

This tool is freely available over the internet on . CMap is free software from the GMOD project .
